# Enhancing the Texture and Sensory Properties of Pickled Cucumbers with Different Brine Solutions

**DOI:** 10.3390/foods14030336

**Published:** 2025-01-21

**Authors:** Mahdieh Yousefi, Akram Arianfar, Vahid Hakimzadeh, Ali Rafe

**Affiliations:** 1 Department of Food Science and Technology, Quchan Branch, Islamic Azad University, Quchan P.O. Box 9479176135, Iran; mahda.yoosefi1268@gmail.com (M.Y.); vahid_hakimzadeh@yahoo.com (V.H.); 2 Department of Food Physics, Research Institute of Food Science and Technology (RIFST), Mashhad P.O. Box 91775-1163, Iran; a.rafe@rifst.ac.ir

**Keywords:** pickle, brine solution, texture, firmness, color, sensory attributes

## Abstract

Softening of pickled cucumbers during storage poses significant challenges for the pickle industry, leading to considerable losses. This softening is attributed to the breakdown of pectic materials in the middle lamella of the cucumber tissue. To address this issue, our study aimed to assess the impact of various ions on cucumber pickle fermentation, storage (over 6 months), as well as overall structure and texture. Fermentation brines were prepared, incorporating different salts, such as KCl, CaCl_2_, MgCl_2_, AlCl_3_, and calcium acetate, at concentrations of 50, 100, 200, and 400 ppm, alongside 6% NaCl. Each month, we removed the pickles from the fermentation brines and analyzed their physicochemical and sensory properties. Our findings revealed that, when pH declined to 3.6, undesirable textural and sensory properties were observed in the pickled cucumbers. However, pickles treated with CaCl_2_ and calcium acetate exhibited higher pH levels compared to other samples after 6 months. Calcium ions demonstrated a positive effect on firmness, contributing to improved consumer acceptance during storage. Among the different salts tested, firmness followed the order of Ca(C_2_H_3_O_2_)_2_ > CaCl_2_ > KCl > MgCl_2_ > AlCl_3_. Furthermore, we observed a positive relationship between pickle firmness and crispness, as well as color values. The sensory evaluation affirmed the positive influence of Ca^2+^ on enhancing pickles’ firmness throughout their shelf-lives. Ca-acetate and CaCl_2_ displayed the most favorable results across sensory, textural, and physical properties among the samples. In conclusion, the addition of Ca-acetate in conjunction with CaCl_2_ was proven to be an effective approach in improving the firmness and extending the shelf-life of cucumber pickles. Our study highlights the potential of calcium ions to enhance the quality and durability of pickled cucumbers, offering promising implications for the pickle industry.

## 1. Introduction

Cucumber (*Cucumis sativus*) pickles are commonly prepared in a brine solution with 1 M (6%) NaCl as a preservative and flavoring agent [1]. Salt serves multiple functions in the fermentation process, such as sustaining firmness and improving the shelf-life of the product up to one year by preventing the effects of degradative enzymes or by reducing bacterial growth [2]. Since softening sometimes—particularly at low NaCl concentrations—occurs during the storage or shelf-life of pickled products, calcium chloride (CaCl_2_) at 20–40 mM is also added to brines to keep the firmness better than NaCl alone [2,3,4,5]. Although a high content of NaCl and CaCl_2_ can prevent both enzymatic and nonenzymatic softening [6], the high level of these ions has undesirable effects on the final product. Therefore, some investigations have also been carried out on salt reduction, which requires blanching prior to fermentation to keep firmness [7].

The interaction between ions such as Ca^2+^ and polyelectrolyte polysaccharides, particularly galacturonans, results in a complex that prevents de-polymerization by hydrolysis [8,9,10]. The bound Ca^2+^ protects against softening that occurs with a lack of softening enzyme activity by avoiding loosening and losses in cohesion of the structural components, rather than by retarding degradation. This has been attributed to changes in galacturonan’s structure, reflected by a decrease in its solubility properties [6]. Different factors, such as dextrose equivalent (DE), ionic strength, and pH, influence the binding of Ca^2+^ to galacturonans [11,12,13]. These galacturonans are quickly de-esterified during fermentation and, therefore, their functional groups and density are not limited by the affinity of Ca^2+^ [6,12,14]. The application of aluminum, which bonds with pectic substances to improve texture, is usually performed during the desalting process, which involves holding in fresh water to reduce NaCl, lactic acid, and other diffusible components in the pickles [15]. Moreover, the pH declines from 5.0 to 3.5 or less, and NaCl immigrates into the tissue to reach the equilibrated concentration at 1 M, developing an acidic condition with high ionic strength that is unfavorable for Ca^2+^ binding [16]. Although the effects of organic acids [17], aluminum in the form of food grade alum (AlK(SO_4_)_2_) [9,10,18], NaCl [2], and calcium [19] on pickle firmness have been investigated in previous studies, the effect of other ions on the binding of galacturonans to maintain the structural integrity, particularly to prolong fermentation and storage, has not been studied. Despite using the best-known good manufacturing practices (GMPs) to retain product quality, the firmness and crispness of pickles gradually decline during storage and marketing. While most fermented pickles should be consumed within a few months, some can be stored for several months prior to use. Undoubtedly, instabilities in texture are a challenge that adversely affects consumer acceptance. Furthermore, consumer acceptance may be adversely affected by products containing salts such as Ca, Mg, and Al, which may be present in some foods and beverages [20].

Moreover, the effect of different cations, including K, Na, Ca, Mg, and Al ions, on the texture and other physical attributes of cucumber pickles has not been evaluated. The monovalent ions Li, K, Rb, and Cs have shown a similar effect to NaCl on the softening of cucumber [10]. Although the effect of calcium salts in the form of chloride, lactate, and acetate on hardness and cross-linking between pectin molecules in black olives has been studied recently [21], and some studies have investigated the effects of chemical treatments—including metal ions, pH, and pectin methylation—on the textural properties of fruits and vegetables [22,23], there has been limited attention on how different cations affect the pH, physicochemical properties, texture, and sensory attributes of cucumber pickles during storage. Therefore, the primary goal of the current study was to investigate the effects of various cations, including K, Na, Ca, Mg, and Al, on the physicochemical, textural, and sensory properties of cucumber pickles during a six-month fermentation and storage period. This research aims to fill a gap in the existing literature by systematically evaluating how different ions influence pH levels, structural integrity, and texture attributes of cucumber pickles. Additionally, the study seeks to examine potential relations between cations and pectin molecules, as well as their impact on the overall sensory acceptance of the pickles. This will provide new insights and practical implications for the pickle industry in addressing the challenges of maintaining texture and quality during storage.

## 2. Materials and Methods

### 2.1. Materials

Fresh pickling cucumbers (size no. 3, measuring 3.8–5.1 cm in diameter and weighing 17–20 g) were purchased from local farms of Shirvan, North Khorasan province, Iran, during June and July 2022. Defect-free cucumbers were weighed (0.4 kg) into plastic containers, covered with brine, and submerged in a 1:1 cucumber to brine ratio. The containers were stored in a dark room at 25 °C and loosely covered with a thin film to avoid possible evaporation.

Fermentation brines were prepared using different salts including KCl, CaCl_2_, MgCl_2_, AlCl_3,_ and calcium acetate, at concentrations of 50, 100, 200, and 400 ppm, along with 6% NaCl. These concentrations constituted the initial cover brine, which also contained 0.15% acetic acid, 1 ppm potassium sorbate, and 0.01% EDTA to lower pH, inhibits yeast and mold growth, and prevents oxidation, respectively. The components within the brine and cucumbers equilibrated to approximately 50% of the initial brine concentrations after the cucumbers were submerged for a few days. Each fermentation treatment was performed witth at least five replicates.

### 2.2. Experiments During the Shelf-Life

Pickles were stored for six months in a dark place at room temperature. Each month, the pickles were removed from fermentation brines, and physicochemical analyses were conducted on the samples.

### 2.3. Brine and Tissue Components

The pH and titratable acidity of the brines and cucumber tissues were measured during the six-month fermentation period according to Buescher’s procedure [15]. The surface color of cucumber pickles was assessed using a black box system, as previously described [24]. Color changes were characterized by values for lightness (L*) and hue angle (calculated as Arctan b/a).

### 2.4. Texture Analysis

The maximum force (N) required to penetrate the cucumber pickles was determined using a TA XT-plus Texture Analyzer (Stable Micro Systems Ltd., Godalming, UK) equipped with a 2 mm diameter blunt tip probe that punctured through 10 mm of mesocarp at a speed of 10 mm/s [9,25]. The texture apparatus had a trigger force of 3 g, and the load cell of 5 kg was also attached to the computer software Stable Micro Systems (version 6.1.14.0, Godalming, Surrey, UK). The whole cucumber was placed horizontally under the probe to ensure equal distance from the endocarp tissues. For each sampling date, three jars of pickles were used, with three pickles measured at different points to assess the penetration force as a measure of firmness. The obtained data were averaged and reported as mean ± SD.

### 2.5. Sensory Assessment

The cucumber pickles were carefully removed from jars at 25 °C for sensory evaluation of crispness by a panel of 20 trained panelists. These individuals were selected based on their ability to detect differences in the crispness of the available cucumber pickles on the market. They received training on how to score the degree of crispness, which was assessed based on a penetration force ranging from 2 to 8 N. Evaluations were performed in silence, fluorescent-lighted booths and included instructions to focus on the degree of crispness. A reference sample of cucumber pickles was selected based on market standards, containing 6% salt in the final product. Panelists were provided with coded samples and instructed to mark their perception of crispness, color, taste, and aroma on a scale from 0 to 10 associated with terms of non (0), very low (1–2), low (3–4), moderate (5–6), high (7–8) and very high (9–10) that had been established during discussions on the parameters ranking. Panelists were also asked to note any detection of off-taste in the samples. The obtained data were averaged and reported as mean ± SD.

### 2.6. Experimental Design and Statistical Analysis

Data from penetration force, pH, acidity, and sensory assessment were analyzed by a two-way analysis of variance (ANOVA), treating storage time and treatment as independent variables. The general linear model procedure was implemented using a statistical software program (Version 9.1, SAS Institute Inc., Cary, NC, USA). Means were compared by Fisher’s least significance difference (LSD) test at *p* < 0.05.

## 3. Results and Discussion

### 3.1. pH and Titratable Acidity Changes During Fermentation

**Figure 1:**
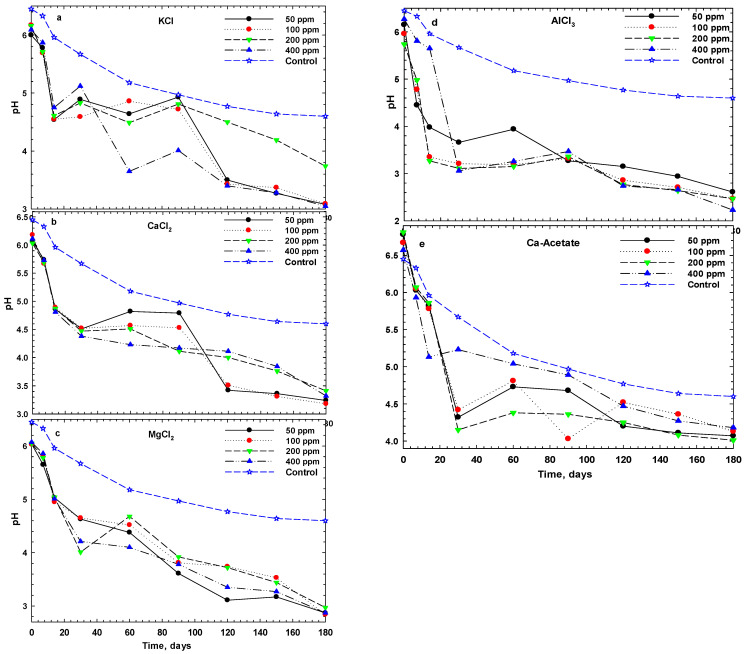
Changes in pH of cucumber pickles with various monovalent (**a**), divalent (**b**,**c**,**e**), and trivalent cations (**d**) salts during fermentation at 25 °C. (Control samples include only NaCl in 1 M concentration and the concentration of salts is in ppm).

The changes in pH of cucumber pickles with various salts, including KCl, CaCl_2_, MgCl_2_, AlCl_3_ and Ca-acetate, at different concentrations, along with control samples (NaCl 1M), during 6 months of storage are given in Figure 1. All salts showed a similar trend in pH changes throughout the storage period. Indeed, pH was decreased during the fermentation storage for all salts. Although the pH dropped from around 6.0 to ~3.5 for all salts, there was a smaller pH reduction (0.5 pH change) in some salts such as calcium acetate and CaCl_2_, while a more significant pH reduction (greater than 1 pH unit) was observed with AlCl_3_ and MgCl_2_ (Figure 1). A comparison of the pH differences between the control and various salts revealed some interesting trends. In the case of divalent cations, there was no significant difference between the pH changes of the control and varying concentrations of salts (Figure 1b,d). However, there was a significant difference for pH changes of the control with AlCl_3_. Although KCl showed minor differences, Ca-acetate exhibited the closest trend to the pH changes of the control. Furthermore, pH declined slowly at the initial storage time, it increased more after 1 month. It appears that the microbial activity in the latter stage of storage, which has also been documented, was mitigated by the acid production caused by fermentation [26]. Similar pH reductions during the first week of fermentation have been previously reported, indicating that high-concentrations of NaCl (8%) significantly decrease pH, stabilizing at around 4.35—an effect attributed to the high salt levels limiting the growth of lactic acid bacteria [27].

Pickled cucumbers with CaCl_2_ and calcium acetate showed higher pH values than other samples after 6 months of storage. This finding implied that the dissociation of calcium salts induced acidification of the brine, which reduces the cucumber’s pH at the initial time of fermentation that is favored for conversion sugars to lactic acid and improved the stability of cucumber pickles containing calcium salts [28,29]. In contrast, the control samples showed a moderate pH, while AlCl_3_, MgCl_2_, and KCl showed the lowest pH values at the end of the storage period. When the pH approached around 3.6, the textural and sensory properties of the cucumbers became unacceptable; therefore, pH can serve as an indicator of pickle shelf life.

**Figure 2:**
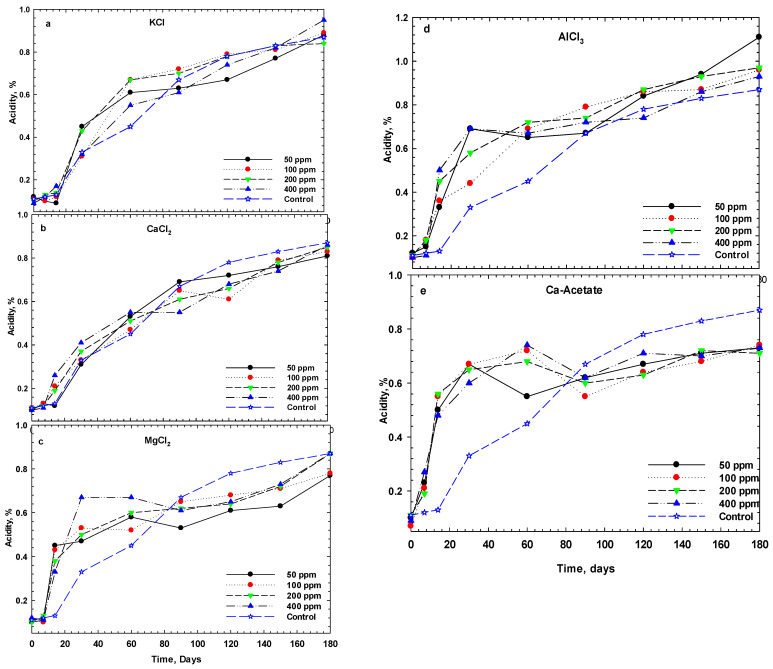
Changes in titratable acidity of cucumber pickles with various monovalent (**a**), divalent (**b**,**c**,**e**), and trivalent cations (**d**) salts during fermentation at 25 °C. (Control samples include only NaCl in 1 M concentration and the concentration of salts is in ppm).

The changes in titratable acidity of cucumber pickles during six months of storage are given in Figure 2. As shown, acidity increased with the decrease in pH during storage, reaching a high level after one month, and thereafter showing minimal fluctuations. The final acidity levels for calcium acetate and CaCl_2_ are lower than those of the other samples, while AlCl_3_, MgCl_2,_ and KCl showed more acidity at the end of storage. Based on the changes in pH and acidity during storage (Figure 1 and Figure 2), it is evident that increasing salt concentration significantly affects both pH and acidity up to a concentration of 100 ppm. However, further increases in salt concentration beyond 100 ppm did not show a significant effect on these parameters. Therefore, we surveyed a comparison among different salts at 100 ppm on pH and acidity alterations in cucumber pickles during 6 months of fermentation.

**Figure 3:**
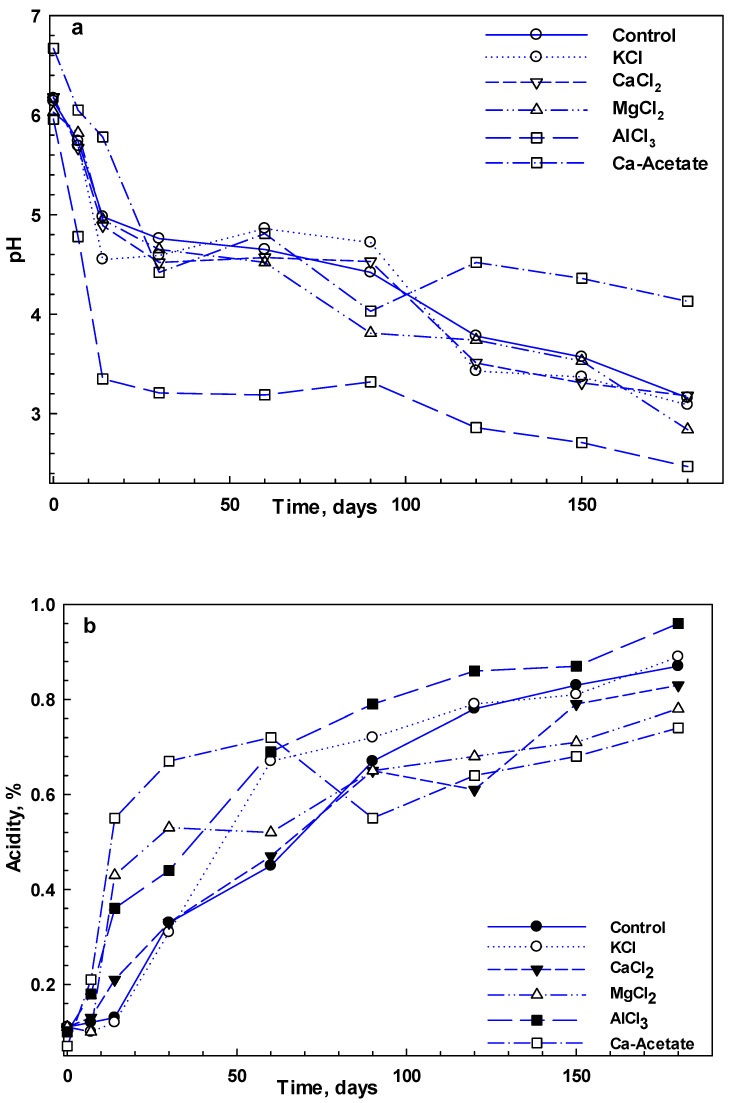
pH (**a**) and acidity (**b**) changes of cucumber pickles including different brine solutions at 100 ppm concentration during 6-month fermentation.

The time course of pH and acidity alterations in cucumber pickles during six months of fermentation is provided in Figure 3. There were significant differences among the different ions, particularly AlCl_3_ and MgCl_2_. As can be seen, pH declined during the fermentation; however, the sharp reduction in pH value occurred in the first week of fermentation. Notably, most pickles’ pH levels fell to 4.6 within a few days after the fermentation began. Similar findings in reducing pH and increasing the fermentation metabolites have been reported for pickle cucumbers with NaCl and CaCl_2_ [28]. The linear regression analysis of pH and acidity across different ion concentrations showed no significant variations. The final pH of fermentations with CaCl_2_ declined from 3.4 to 3.0 as the CaCl_2_ concentration increased, even though the production of acids did not change. This phenomenon may be related to the dissociation constants of carboxylic acids, which tend to increase with higher ionic strength [30]. The average pH of the brine solutions—with the exception of acetate and AlCl_3_—was measured at 3.74 ± 0.21 after 4 months, although after 4 months of storage, the pH of Ca-acetate and AlCl_3_ remained approximately at 4.0 and below 2.7, respectively.

### 3.2. Textural Properties

**Figure 4:**
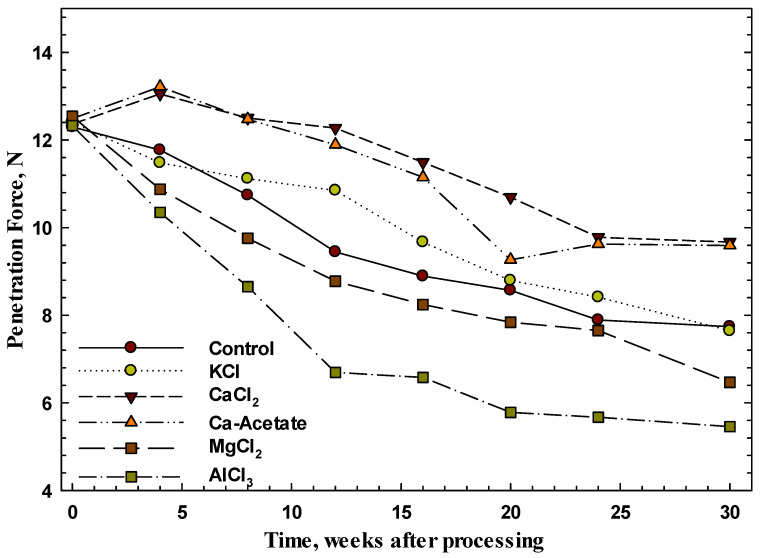
Changes in firmness (N) during fermentation of pickle cucumber affected by different ions at 100 ppm concentration.

Maximum penetration force (N) is considered as firmness, which correlates well with sensory evaluation [31]. The force required to penetrate the mesocarp tissue of cucumber pickles during six months of fermentation is provided in Figure 4. The firmness of all samples was decreased during storage which was caused by reductions in and the redistribution of ions [6]; however, this reduction notably occurred in the second month for the Al^3+^ and Mg^2+^.

The mean peak force of fresh cucumber pickles before fermentation was about 12 N across all treatments; however, this value decreased during fermentation. These firmness values were rather higher than those reported in the previous work [32], which may be related to the type of cucumber origin and the content of salt brine solution. Moreover, firmness significantly declined in all treatments during storage, except for CaCl_2_ and Ca-acetate, which exhibited more firmness after 30 weeks of storage. It has also been reported that the firmness of cucumber pickles after processing and during fermentation was not changed even in comparison to the firmness before fermentation [9]. Additionally, Ca^2+^ exhibited greater firmness during fermentation, consistent with findings that CaCl_2_ reduces the softening of cucumber pickles during storage [5,6,9,15]. Firmness and resistance to softening induced by Ca have been attributed to the formation of Ca-pectates that increase middle lamella cell wall rigidity and resistance to degradation by polygalacturonase. It has been previously observed that the protection against mesocarp softening during storage occurrs when the brine contains 700 ppm Ca^2+^ or 300 ppm Ca^2+^ along with 75 ppm Al^3+^ [9,15,28]. The pickled cucumbers brined in NaCl (control) showed an average firmness of 8 N, while the Ca^2+^-brined counterparts had significantly higher firmness (~10 N) (*p* < 0.05), and similar results have been reported elsewhere [28,32]. The firmness of the MgCl_2_ samples after 30 weeks of fermentation was about 7 N, which showed a positive relation and was similar to the bay salts [33]. Accordingly, the order of firmness after six months of storage for different salts is as follows: Ca(C_2_H_3_O_2_)_2_ > CaCl_2_ > NaCl > KCl > MgCl_2_ > AlCl_3_. In addition, the firmness of all samples was increased by increasing the salt concentration, which may be attributed to the interactions between galacturonic acid and the ions, as previously reported for different concentrations of CaCl_2_ [28]. By considering the constant amount of galacturonic acid in the pickled cucumber (300 ppm) [6], the stoichiometric effects of the salts can be evaluated. Firmness of cucumber pickles is closely associated with the solubility characteristics and degree of esterification (DE) of pectic substances [33]. Moreover, the effect of thermal and fermentation on the texture of cucumber pickles have been studied, and it has been understood that the thermal degradation of firmness and crispness in fermented cucumbers follows a first-order process [31].

### 3.3. Sensorial Properties

Since the firmness of pickles was previously shown to be correlated with sensory attributes such as crunchiness and crispness [9,14], the relationship between sensory properties and textural results was investigated. Firmness and crispness of the cucumber pickles are crucial critical textural attributes that show the good quality of the product. It has been found that these factors are major parameters affecting the consumer acceptance of cucumber pickles [6]. Therefore, these critical textural properties, along with color, aroma, and taste, were examined in the 3rd and 6th months of storage, as fermentation is completed within two months (see Section 3.1).

**Figure 5:**
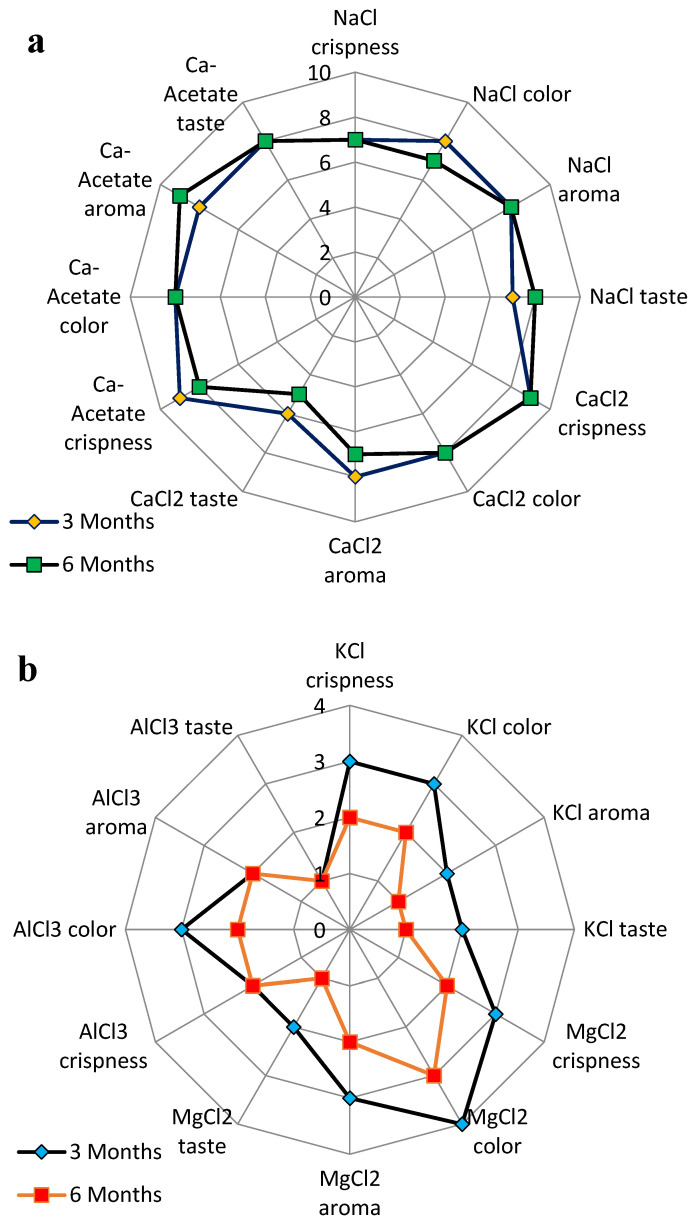
Sensory scores of the cucumber pickles including crispness, aroma, color, and taste during storage at 3rd (**a**), and 6th months (**b**) for different brine solutions at 100 ppm of concentration.

**Figure 6:**
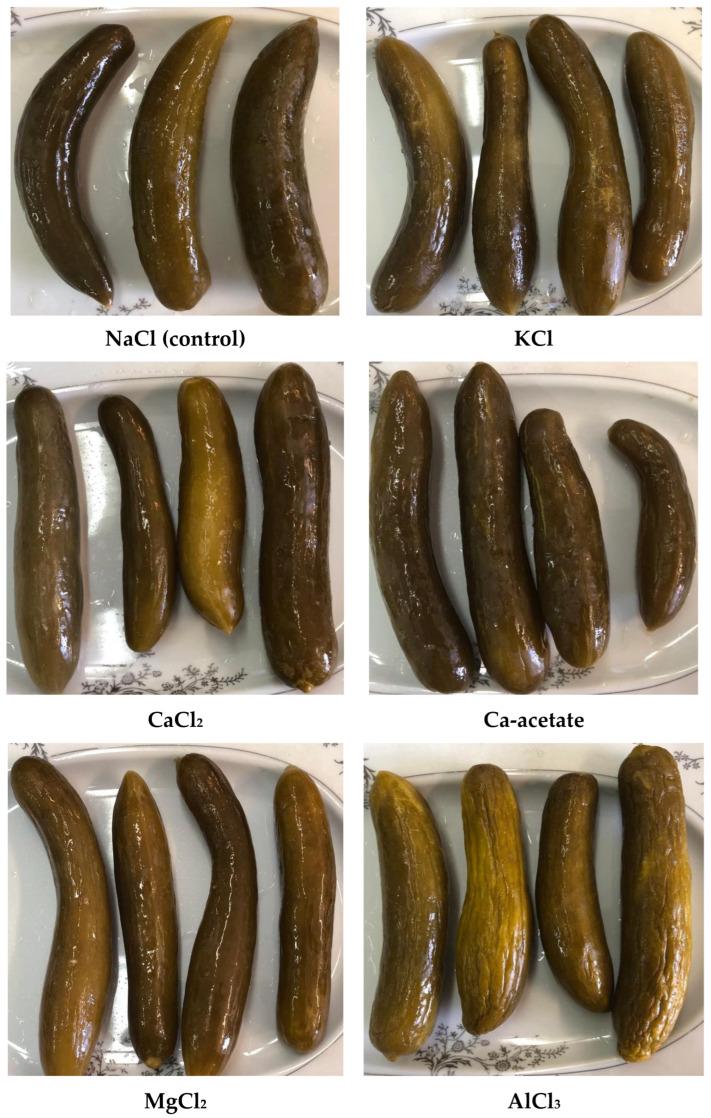
Pictures of cucumber pickles as affected by different salt brine after six months of storage.

It can be seen that the firmness and crispness of all samples were affected by the type of salts (Figure 5). The high crispness was obtained for the samples containing CaCl_2_ or Ca-acetate, while the lower values were achieved for KCl, MgCl_2,_ and AlCl_3_ (Figure 5a,b). Similarly, the taste of the treatments was not the same and the aroma and color were affected more by KCl, MgCl_2,_ and AlCl_3_. As can be seen in Figure 6, the cucumber pickles containing AlCl_3_ experienced shrinkage and discoloration. Moreover, the taste, aroma, and color of KCl and MgCl_2_ were not satisfactory as evaluated by the consumers. Therefore, KCl, MgCl_2,_ and AlCl_3_ solutions cannot be considered for cucumber pickles, while CaCl_2_ and Ca-acetate had the best scores from the trained panelists. It has been reported that the ability of calcium to maintain cucumber firmness at low pH is related to its capacity to bind with cell wall polysaccharides. The maximum concentration of Ca^2+^ in pickled products is less than 25 mM due to its chalky flavor at high concentrations, which is undesirable in the final product. Since calcium can bind to plant cell walls, it is more of interest to determine which calcium salts in the fermented cucumbers would readily be accepted by the consumers. According to Figure 5a, it can be understood that Ca-acetate received higher scores in sensory evaluation, which may be related to the acidic condition of the brine solution, and therefore, the aroma and taste were improved by Ca-acetate. In the view of the panelist, Ca-acetate, CaCl_2_, and NaCl achieved the best scores, correlating well with their textural properties.

It has been also found that the crispness of cucumber pickles has a positive relation with the sensory properties, which is in line with our study [31]. Furthermore, the sound generated from chewing pickles plays a major key in the perception of its texture and can be used to evaluate its textural properties. Therefore, a penetration test of cucumber pickles is an effective way of measuring pickle crispness. Some studies have also evaluated the sounds of chewing cucumber pickles and stated their positive relationship with crispness [1]. Accordingly, it has been understood that the sounds associated with texture and particularly crispness can be applied to cucumber pickle quality [1,31]. The maximum crispness and retention of the crispness were achieved when cucumbers were fermented in brines of Ca-acetate and CaCl_2_.

### 3.4. Color Attributes

**Figure 7:**
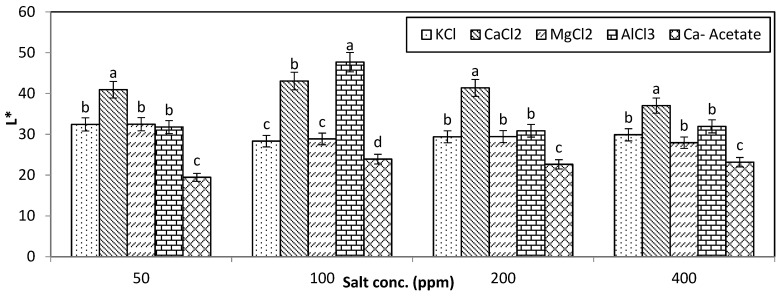

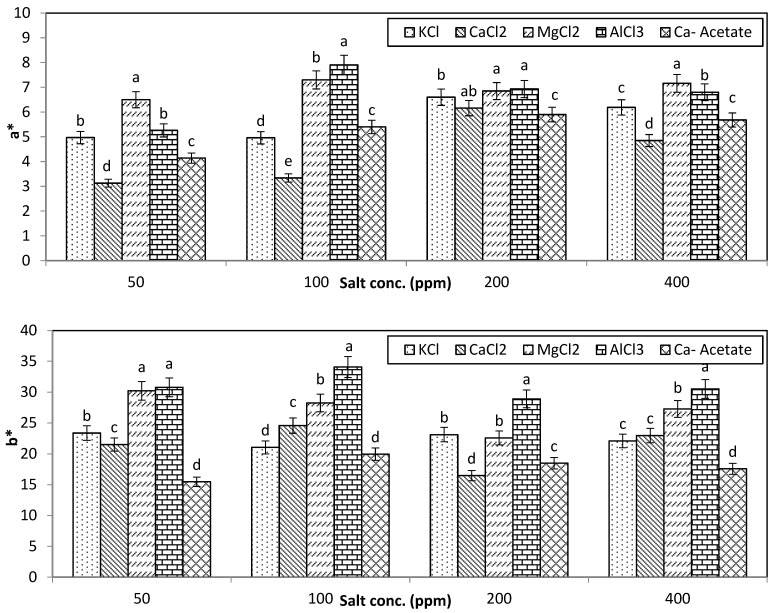
Effect of brine type and concentrations on the color attributes of cucumber pickles after 6 months of storage. The statistically significant difference among the samples at each concentration is provided by alphabetic letters (*p* < 0.05).

The L*a*b* values of cucumber pickles at three and six months in different brine solutions are presented in Figure 7. The highest L* (lightness) values of the samples were observed in CaCl_2_ solution, while the lowest values were obtained for Ca-acetate. There were no significant differences among the other samples (*p* < 0.05). According to Lab values and Figure 6, all fermented pickles exhibited a dark olive-green surface color. The presence of CaNa_2_EDTA is known to reduce the L* value, particularly at high level of EDTA [8]. Therefore, it can be concluded that storage conditions had an impact on the L* values, likely due to the salts of EDTA. Specifically, the darker olive-green color seen in all fermented samples may have impacted their perceived freshness and appeal. The maximum a* values (redness) and b* values (yellowness) were obtained for AlCl_3_ and MgCl_2_, although there were not any significant differences among the other samples (*p* < 0.05). However, these color attributes did not translate into favorable sensory evaluations, as the KCl, MgCl_2_, and AlCl_3_ treatments were noted for their inadequate taste and aroma profiles, ultimately resulting in lower consumer acceptability. Furthermore, the hue angle was in the range of 1.21–1.43 for all cucumber pickles and the maximum and minimum values of hue angle were seen for CaCl_2_ (1.43) and NaCl (1.21) and Ca-acetate (1.22), respectively. There were no significant differences among the other samples (*p* < 0.05). Some researchers also investigated the color of the pickle and reported similar results which are in line with our results [34,35].

In conclusion, while the sensory analysis highlighted the superiority of CaCl_2_ and Ca-acetate in terms of textural attributes, the color analysis indicated that the L*, a*, and b* values were also critical in shaping consumer perceptions. Thus, a comprehensive evaluation of both color and sensory characteristics is essential for optimizing pickle quality and consumer satisfaction.

## 4. Conclusions

This study uncovers significant insights into the effects of various salt solutions on the preservation of cucumber pickles, particularly highlighting the roles of calcium salts in maintaining firmness and sensory quality during extended storage. While softening in pickles are a well-documented challenge, our findings reveal that the strategic application of CaCl_2_ and calcium acetate not only mitigates this issue but also enhances the overall shelf-life of pickled products. Interestingly, we discovered that the higher pH levels associated with calcium salt treatments promote desirable textural and sensory qualities, suggesting a potential avenue for optimization in commercial pickling processes. Our results indicate that a pH threshold of around 3.6 may serve as a predictive marker for acceptable firmness and taste profiles. This knowledge provides food technologists with a valuable tool for assessing pickle quality over time.

Moreover, the mechanistic insights gained from observing the formation of Ca-pectates, which enhance cell wall integrity, open new research pathways to explore additional compounds or methods that could further reinforce this protective effect in food products. The superior sensory acceptance of Ca-acetate and CaCl_2_ compared to traditional salt solutions like KCl, MgCl_2_, and AlCl_3_ invites a re-evaluation of brining practices, particularly for enhancing texture without compromising flavor or color attributes. In light of consumer preferences for freshness and texture, our study emphasizes the importance of calcium ion concentrations, particularly 100 ppm in conjunction with 1 M NaCl, as a promising strategy for extending the shelf-life of cucumber pickles while preserving their quality. Future research should focus on exploring the long-term effects of these brine solutions in different environmental conditions and their potential interactions with other natural additives, thereby broadening our understanding of optimal pickling techniques. 

## Data Availability

The original contributions presented in the study are included in the article; further inquiries can be directed to the corresponding author.
